# Topical Drug Delivery: Innovative Controlled Release Systems

**DOI:** 10.3390/pharmaceutics15061716

**Published:** 2023-06-13

**Authors:** Daniele Ribeiro de Araujo, Cristina Padula

**Affiliations:** 1 Human and Natural Sciences Center, ABC Federal University, Santo Andre 09210-580, SP, Brazil; 2 Drugs and Bioactives Delivery Systems Research Group—SISLIBIO, ABC Federal University, Santo Andre 09210-580, SP, Brazil; 3 Department of Food and Drug, University of Parma, Parco Area delle Scienze, 27/a, 43124 Parma, Italy

One of the most innovative strategies for administrating bioactive molecules is the design of adequate drug delivery systems. In parallel, researchers have also devoted their efforts towards alternative routes of administration to increase patient compliance and reduce side effects. In this sense, the topical route is convenient compared with traditional routes due to self-application, non-invasive administration, local drug release and avoiding first-pass biotransformation. However, the topical route includes drug administration in different morphologically structured tissues with their particularities in cellular organization and membrane composition, such as the skin and mucosa, which requires the development of well-designed nanocarriers to achieve improvements in drug permeation, adequate drug concentrations into the site of action and easy application. Herein, all studies explored trends in skin and mucosa structural evaluation, the role of new excipients as permeation enhancers, in vitro/in vivo correlations and drug delivery from different nanocarriers and matrices.

In fact, the formulation composition is essential to determine the extent of topical and transdermal drug permeation. Then, [1] provided an extensive view on both drugs and vehicles permeation, with in vitro–in vivo relationships. The insights from those results revealed new approaches on development of effective and safer skin formulations.

In addition to drug permeation, authors also described innovative strategies to improve drugs skin retention and reducing at the same time transdermal flux. In [2], authors focused their studies on specific application of imiquimod-loaded micelles for the treatment of actinic keratosis, by associating co-solubilizers agents such as oleic acid. On the other hand, the use of hydrophilic nanocarriers has been reported for similar purposes, as described by authors [3]. This study considered the treatment of atopic dermatitis by complexing budesonide with cyclodextrin and its further incorporation into poloxamer-based hydrogels. The main findings reported that the drug solubility into the matrix was essential to enhance the retention in skin layers.

Microneedle technologies were addressed in two works. [4] evaluated the imiquimod skin delivery and proposed the application of these systems for the treatment of warts. This theme was also discussed in the review by [5]. The authors proposed new groups of chemical permeation enhancers including lipid synthesis inhibitors, cell-penetrating peptides and ionic liquids, which are described in terms of chemical structure and main applications as well as their molecular mechanisms of action and the consequences on drug permeation improvement after incorporation in different types of microneedles.

The use of chemical permeation enhancers was also reported by [6]. In an experimental approach, authors evaluated the performance of caprylic acid or sodium taurocholate for promoting the permeation of high-molecular-weight dextrans across the buccal mucosa. A description of each successful result is provided, considering the mucosa morphological particularities.

Other innovative technologies were widely addressed in this Special Issue, including niosomes, liposomes and gels matrices. Structural organization, skin delivery performance and permeation mechanisms were assessed, aiming to find different anti-cancer, hormone replacement and skin inflammatory lesions therapies. [7] loped a niosome-loaded *Aspergillus oryzae*-fermented soybean extract for improving estrogen skin absorption with considerable advantages regarding the reduction of systemic side effects. In another study, [8] luorouracil-loaded aptamer-conjugated liposomes and their further incorporation into sodium alginate/hyaluronic acid gels, looking to have a new therapeutic tool against basal cell carcinoma. The production of new gels was also reported by [9] a thermosensitive organogel based on isopropyl myristate-soy lecithin and poloxamers as oil and aqueous phase, respectively, for dual drug delivery, producing highly structured gels that lead to the stratum corneum lipids transitioning from a hexagonal to a liquid crystal phase. Finally, the incorporation of lipid components into a hydrogel matrix was reported by [10]. when comparing different compositions of a chrysin-loaded nanoemulsifying system for melanoma-affected skin treatment. The enhancement of drug solubility, therapeutic effects and the purpose of new formulations with innovative compositions were the main findings explored.

This Special Issue covered relevant aspects of innovations and technological tools applied to topical drug administration. The broad application of all studies highlighted that the search for effective, safe and innovative skin formulations need a growing renewal of knowledge based on the extensive work, from the initial idea until the optimization of the manufacturing process and their possible scale-up aspects. Taken together, the scientific and technological contributions of this Special Issue drive the creation of a nanotechnological approach in the development of effective and biocompatible pharmaceutical formulations, which motivated the selection of components and bioactive molecules discussed herein.

## Data Availability

Not applicable.
